# Optimized Lightweight Architecture for Coronary Artery Disease Classification in Medical Imaging

**DOI:** 10.3390/diagnostics15040446

**Published:** 2025-02-12

**Authors:** Akmalbek Abdusalomov, Sanjar Mirzakhalilov, Sabina Umirzakova, Ilyos Kalandarov, Dilmurod Mirzaaxmedov, Azizjon Meliboev, Young Im Cho

**Affiliations:** 1 Department of Computer Engineering, Gachon University Sujeong-Gu, Seongnam-si 13120, Republic of Korea; akmaljon@gachon.ac.kr; 2 Department of Computer Systems, Tashkent University of Information Technologies Named After Muhammad Al-Khwarizmi, Tashkent 100200, Uzbekistan; mirzaxalilov86@tuit.uz; 3 Department of Automation and Control, Navoi State University of Mining and Technologies, Navoi 210100, Uzbekistan; kalandarovilyos1987@gmail.com; 4 Department of Artificial Intelligence, Tashkent State University of Economics, Tashkent 100066, Uzbekistan; d.mirzaakhmedov@tsue.uz; 5 Department of Digital Technologies and Mathematics, Kokand University, Kokand 150700, Uzbekistan; a.meliboyev@kokanduni.uz

**Keywords:** coronary artery disease, athletes, lightweight model, cardiovascular diagnostics, medical imaging

## Abstract

**Background/Objectives**: The early and accurate detection of Coronary Artery Disease (CAD) is crucial for preventing life-threatening complications, particularly among athletes engaged in high-intensity endurance sports. This demographic faces unique cardiovascular risks, as prolonged and intense physical exertion can exacerbate underlying CAD conditions. Studies indicate that while athletes typically exhibit enhanced cardiovascular health, this demographic is not immune to Coronary Artery Disease (CAD) risks. Research has shown that approximately 1–2% of competitive athletes suffer from CAD-related complications, with sudden cardiac arrest being the leading cause of mortality in athletes over 35 years old. High-intensity endurance sports can exacerbate underlying CAD conditions due to the prolonged physical stress placed on the cardiovascular system, making early detection crucial. This study aimed to develop and evaluate a lightweight deep learning model for CAD detection tailored to the unique challenges of diagnosing athletes. **Methods**: This study introduces a lightweight deep learning model specifically designed for CAD detection in athletes. By integrating ResNet-inspired residual connections into the VGG16 architecture, the model achieves a balance of high diagnostic accuracy and computational efficiency. By incorporating ResNet-inspired residual connections into the VGG16 architecture, the model enhances gradient flow, mitigates vanishing gradient issues, and improves feature extraction of subtle morphological variations in coronary lesions. Its lightweight design, with only 1.2 million parameters and 3.5 GFLOPs, ensures suitability for real-time deployment in resource-constrained clinical environments, such as sports clinics and mobile diagnostic systems, where rapid and efficient diagnostics are essential for high-risk populations. **Results**: The proposed model achieved superior performance compared to state-of-the-art architectures, with an accuracy of 90.3%, recall of 89%, precision of 90%, and an AUC-ROC of 0.912. These metrics highlight its robustness in detecting and classifying CAD in athletes. The model lightweight architecture, with only 1.2 million parameters and 3.5 GFLOPs, ensures computational efficiency and suitability for real-time clinical applications, particularly in resource-constrained settings. **Conclusions**: This study demonstrates the potential of a lightweight, deep learning-based diagnostic tool for CAD detection in athletes, achieving a balance of high diagnostic accuracy and computational efficiency. Future work should focus on integrating broader dataset validations and enhancing model explainability to improve adoption in real-world clinical scenarios.

## 1. Introduction

CAD remains one of the leading causes of morbidity and mortality worldwide, posing significant challenges to effective detection and management [1]. Among various population groups, athletes engaged in high-intensity and high-volume endurance sports face unique cardiovascular risks [2]. While this demographic is typically associated with enhanced cardiovascular health, prolonged physical exertion inherent to their activities can exacerbate underlying CAD conditions [3]. This often asymptomatic presentation increases the likelihood of severe complications, including sudden cardiac arrest, particularly in athletes over the age of 35 [4]. These factors underscore the critical need for early and accurate CAD diagnosis tailored to this high-risk population [5]. These challenges are further compounded by the often asymptomatic presentation of CAD in this population, necessitating the development of advanced diagnostic tools capable of early and accurate detection [6]. In this context, the integration of deep learning (DL) techniques into medical imaging presents a transformative opportunity [7]. Deep learning has demonstrated exceptional performance in image-based classification tasks, particularly in the domain of medical diagnostics [8]. Among the numerous DL architectures, VGG16 has emerged as a robust and well-established baseline for image classification [9]. Conventional methods for CAD diagnosis, such as invasive coronary angiography, remain the gold standard but are resource-intensive and associated with patient risks [10]. Non-invasive imaging modalities, while safer, often rely on manual interpretation that is prone to observer bias and demands significant expertise [11]. While existing DL architectures such as ResNet, DenseNet, and EfficientNet have demonstrated exceptional performance in medical imaging, their computational complexity limits their suitability for real-time clinical deployment, especially in resource-constrained environments like sports clinics or mobile diagnostic systems [12]. However, its limitations, particularly in addressing vanishing gradient issues and leveraging deeper feature representations, highlight the need for further architectural enhancements tailored to the complexities of medical data [13]. To address these limitations, this study proposes a novel lightweight deep learning model specifically designed for CAD detection in athletes. By incorporating ResNet-inspired residual connections into the VGG16 architecture, the model enhances gradient flow, mitigates vanishing gradient issues, and improves feature extraction of subtle morphological variations in coronary lesions. This approach improves diagnostic accuracy and ensures computational efficiency, enabling real-time application in resource-limited settings. The main contributions of this study are as follows:

- Modifying the VGG16 model with ResNet-inspired residual connections, tailored specifically for CAD detection in athletes, proposes a novel lightweight architecture.
- The model achieves superior diagnostic performance compared to state-of-the-art architectures, with significantly reduced computational complexity.
- Comprehensive evaluation using the CADICA dataset demonstrates its effectiveness across key metrics, including accuracy, recall, and AUC-ROC.
- Practical implications for deploying the model in real-time diagnostic workflows in sports medicine are discussed.

This innovation is particularly critical in the context of CAD, where subtle variations in lesion morphology and severity necessitate high sensitivity and specificity in feature extraction. Central to this work is the use of the CADICA dataset, a comprehensive collection of 3000 grayscale angiographic images specifically curated to represent varying severities of coronary artery lesions. The dataset is meticulously annotated into three categories—‘Mild’, ‘Moderate’, and ‘Severe’—with corresponding lesion severity ranges. To simulate real-world diagnostic conditions, the dataset incorporates image augmentations reflecting variations in imaging angles, lighting conditions, and anatomical diversity. This ensures the robustness and generalizability of the proposed model in diverse clinical scenarios. Through a rigorous experimental framework, the proposed model demonstrates superior performance across key evaluation metrics, including accuracy, recall, and precision, surpassing state-of-the-art architectures such as ResNet-50, DenseNet121, and EfficientNet-B0. The proposed modifications not only enhance the model capacity for nuanced feature representation but also reduce computational inefficiencies, making it a viable candidate for real-time diagnostic applications. The findings of this research underscore the transformative potential of integrating advanced deep learning methodologies into cardiovascular diagnostics. By addressing the unique challenges posed by CAD in athletes, this work contributes to a broader understanding of AI-driven personalized healthcare solutions. Moreover, the proposed framework paves the way for future research aimed at enhancing diagnostic precision and efficiency, ultimately improving patient outcomes in high-risk populations.

The remainder of this paper is organized as follows: Section 2 reviews related works on CAD detection and deep learning models. Section 3 presents the proposed methodology, including architectural innovations. Section 4 discusses the dataset, experimental setup, results, and analysis. Section 5 highlights key findings, limitations, and future research directions, while Section 6 concludes the study.

## 2. Related Works

The application of deep learning (DL) in medical imaging has witnessed significant advancements over the past decade [14], transforming diagnostic workflows and enabling more precise detection of diseases such as CAD [15]. Numerous studies have explored the integration of convolutional neural networks (CNNs) into CAD diagnosis, focusing on enhancing both detection accuracy and computational efficiency [16]. This section reviews the key contributions of prior research in CAD detection, deep learning architectures, and dataset utilization.

### 2.1. Traditional Methods for CAD Diagnosis

Historically, CAD diagnosis relied heavily on invasive techniques, such as coronary angiography, which remain the gold standard but are resource-intensive and associated with patient risks. The emergence of non-invasive imaging modalities, including computed tomography (CT) and magnetic resonance imaging (MRI), offered safer alternatives; however, their manual interpretation is prone to observer bias and requires significant expertise [17]. To mitigate these limitations, computer-aided diagnosis (CADx) systems were introduced, utilizing conventional machine learning algorithms like Support Vector Machines (SVMs) [18] and Random Forests [19]. Ref. [20] has evaluated seven computational feature selection (FS) methods, a domain knowledge-based FS method, and various classification algorithms to enhance CAD diagnosis. In [21], ML models were developed to predict CAD presence and assess severity. The eXtreme Gradient Boosting (XGBoost) model showed the highest efficacy for predicting CAD, achieving an area under the receiver operating characteristic curve of 0.728 (95% CI: 0.623–0.824), with significant predictors including left ventricular ejection fraction, homocysteine, and hemoglobin (*p* < 0.001). Additionally, XGBoost effectively predicted CAD severity, correlating SYNTAX and GENSINI scores with factors such as brain natriuretic peptide, glycated hemoglobin, and high-density lipoprotein (*p* < 0.001). Ref. [22] study proposed a non-invasive method for CAD diagnosis using iris images, combining iridology with advanced image processing techniques. This method analyzed the iris of 198 participants, including 94 CAD patients and 104 healthy controls. The iris was preprocessed using integral differential operator and rubber sheet methods, and features were extracted through wavelet transforms and statistical analyses such as Gray-Level Co-Occurrence Matrix (GLCM) and Gray-Level Run-Length Matrix (GLRLM). While these early systems demonstrated potential, their reliance on handcrafted features limited their adaptability and performance in complex medical datasets.

### 2.2. Deep Learning in Medical Imaging

Deep learning has revolutionized medical imaging by automating feature extraction and improving classification accuracy. Architectures such as AlexNet, ResNet, and VGG have been widely adopted in various diagnostic applications, including lung cancer detection, brain tumor segmentation, and diabetic retinopathy classification [23]. For CAD diagnosis, studies leveraging CNNs have demonstrated their ability to identify subtle morphological changes in coronary arteries that may elude manual inspection [24]. For instance, ResNet-based models have shown promise in detecting stenosis in coronary angiograms due to their use of residual connections, which alleviate vanishing gradient problems in deep networks [25]. Similarly, studies employing DenseNet have highlighted the advantages of densely connected layers for feature reuse, leading to more compact and efficient models [26]. Ref. [27] evaluated a DL-based imaging reconstruction and diagnosis system designed to enhance diagnostic accuracy and efficiency. The study involved 374 cases across five sites, comparing the diagnostic performance of radiologists using traditional methods versus those assisted by the DL system, with invasive coronary angiography serving as the reference standard. Ref. [28] evaluated segmentation models (U-Net, ResUNet-a, and UNet++) and classification models (DenseNet201, EfficientNet-B0, MobileNet-v2, ResNet101, and Xception) for this purpose. Among the segmentation models, U-Net outperformed others with a Dice score of 0.8467 and a Jaccard Index of 0.7454. Ref. [29] introduced PlaqueNet, a segmentation model for coronary plaques in CCTA images. The model achieved exceptional segmentation performance by integrating a deep-wise residual optimization module and DASPP-BICECA for enhanced feature sensitivity and broader information capture. However, these architectures are not without limitations, particularly in terms of computational requirements and the need for large, annotated datasets. In response to the challenges of standard CNN architectures, researchers have explored various modifications to enhance their applicability in CAD detection [30]. For example, integrating attention mechanisms has been proposed to improve the localization of lesions in angiographic images, while multi-scale feature extraction methods have been employed to capture both global and local context [31]. Additionally, hybrid models combining CNNs with recurrent neural networks (RNNs) have been introduced to analyze temporal variations in sequential imaging data, further enriching diagnostic insights [28]. Ref. [32] proposed O-SBGC-LSTM, enhanced with the Eurygaster Optimization Algorithm (EOA), to improve early detection. This model captures spatial-temporal features and uses a hierarchical architecture to boost accuracy (>98%) while reducing computation costs. Coronary artery stenosis detection is inherently challenging due to complex vascular structures, low-quality imaging, and artifacts caused by breathing or stenotic lesions. The Ref. [33] DCA-YOLOv8 framework addressed these issues through advanced modules, including histogram equalization and canny edge detection preprocessing (HEC), double coordinate attention (DCA) feature extraction module, and a novel adaptive inner-CIoU (AICI) loss function. Despite these advancements, the reliance on computational resources and extensive data highlights the need for further optimization and innovation in deep learning methods for CAD diagnosis. The VGG16 architecture, widely regarded for its simplicity and effectiveness, has been a frequent baseline in medical imaging research [34]. Studies modifying VGG16 to include residual blocks or attention layers have demonstrated significant improvements in performance metrics, such as sensitivity and specificity, particularly for challenging tasks like CAD classification [35]. These modifications underscore the potential of adapting existing architectures to address domain-specific requirements.

These models demonstrate that lightweight architectures can achieve high performance while minimizing computational costs, making them ideal for deployment in constrained settings. However, most lightweight architectures are developed for generic image recognition tasks and are not tailored to domain-specific challenges such as CAD detection. For example, coronary angiography images require models capable of detecting subtle variations in lesion morphology, which demand a balance between sensitivity and computational efficiency. Our work builds upon the foundational principles of lightweight architectures by proposing a novel modification to VGG16. By integrating ResNet-inspired residual connections, we enhance the model’s ability to extract nuanced features critical for CAD detection while maintaining a lightweight design with only 1.2 million parameters and 3.5 GFLOPs. This makes our model particularly well-suited for real-time applications in resource-constrained environments, such as sports clinics, where rapid and accurate diagnostics are essential.

### 2.3. Datasets for CAD Research

The quality and diversity of datasets play a pivotal role in training robust DL models. Publicly available datasets such as DRIVE [36] and STARE [37] have facilitated research in ophthalmology, while datasets like LIDC-IDRI [38] have been instrumental in lung cancer detection. For CAD, datasets are often limited in size and scope, posing challenges to model generalization. The CADICA dataset [28] used in this study addresses this gap by providing a balanced and well-annotated collection of angiographic images, categorized by lesion severity. This dataset’s inclusion of augmented images simulating real-world conditions further enhances its utility for training and evaluating DL models.

### 2.4. Evaluation Metrics and Benchmarking

Performance evaluation of CAD detection models typically involves metrics such as accuracy, recall, precision, and F1-score. Studies have emphasized the importance of balancing sensitivity and specificity, given the high stakes of false negatives and false positives in medical diagnostics. Additionally, computational efficiency, measured through parameters such as Floating Point Operations Per Second (FLOPs) and inference time, has become an increasingly important consideration for real-time applications.

### 2.5. Gaps and Challenges in Current Researches

Despite the progress in DL-based CAD detection, several gaps remain. The reliance on limited datasets restricts model generalization, while the computational complexity of advanced architectures hinders their deployment in resource-constrained settings. Furthermore, the lack of interpretability in DL models presents challenges for clinical adoption, as medical practitioners require transparent and explainable systems to trust their outputs. Addressing these challenges through innovative architectural modifications, such as the integration of residual connections in this study, represents a critical step forward. Building on the foundation of prior research, this study advances the state of the art in CAD detection by introducing a modified VGG16 architecture tailored to the unique requirements of angiographic image analysis. By leveraging the strengths of residual learning and addressing the limitations of existing approaches, this work contributes to the growing body of knowledge in AI-driven cardiovascular diagnostics.

## 3. Methodology

The core idea of this work is to propose a lightweight architecture by modifying the VGG16 model, tailored for CAD detection in athletes. While the integration of ResNet-inspired blocks is not entirely novel, our approach is unique in its emphasis on lightweight design, achieved through a significant reduction in trainable parameters and computational complexity. This is particularly critical for real-time applications in resource-constrained environments, such as mobile diagnostic systems and sports clinics. In this paper, we introduce an advanced methodology for the classification of CAD among athletes engaged in high-intensity and high-volume endurance sports. This demographic presents unique challenges and requires specialized approaches for effective management. Our method leverages a modified version of the deep learning model VGG16, tailored specifically for this application. The early detection and prevention of CAD through systematic health screenings and heightened awareness of its symptoms are critical, particularly as athletes age or escalate the intensity of their training routines. In Section 3.1 of our paper, we meticulously outline the original architecture of the baseline model. Following this, Section 3.2 details our proposed model modifications, providing a comprehensive framework for understanding our innovative approach.

### 3.1. VGG16

The VGG16 architecture, a prominent deep learning model, was developed by researchers from the Visual Graphics Group at Oxford, from which it derives its name. Designed to enhance image classification tasks, this model achieved significant recognition for its performance, particularly in the ImageNet competition. The architecture comprises sixteen layers with trainable parameters, including thirteen convolutional layers and three fully connected layers. At its core, the convolutional layers employ filters with a small receptive field of 3 × 3, which is the minimal size capable of capturing spatial information such as left, right, up, down, and center. To preserve the spatial resolution of input images, these layers use a stride fixed at 1 pixel and padding of 1 pixel. The max pooling layers, interspersed between the convolutional layers, utilize a 2 × 2 pixel window with a stride of 2 to progressively reduce the spatial dimensions while retaining the most significant features. The number of filters in the convolutional layers begins at 64 in the initial layer and doubles after each max pooling operation, scaling to 128, 256, and eventually 512 filters. This gradual increase allows the model to capture more complex and hierarchical features at deeper layers. The activation function applied throughout the network is the Rectified Linear Unit (ReLU), which introduces non-linearity, enabling the model to learn intricate patterns. After passing through the convolutional and pooling layers, the data is processed by three fully connected layers. The first two layers each consist of 4096 channels, while the final layer, designed for classification, performs a 1000-way classification and outputs a probability distribution over 1000 class labels using a softmax activation function. Training the VGG16 network involves backpropagation with a cross-entropy loss function, also known as multinomial logistic regression loss, applied to the output of the softmax layer. The architecture’s uniformity, characterized by its consistent use of small, 3 × 3 filters, makes it straightforward to understand and adapt for various tasks. However, its simplicity comes at a cost, as the network is computationally intensive and requires significant memory resources due to its large number of parameters. Despite these challenges, VGG16 remains a foundational model, valued for its ability to extract complex features and its adaptability to a wide range of applications.

### 3.2. The Proposed Model

The proposed model is a lightweight architecture tailored for CAD detection in athletes by modifying the VGG16 architecture. While incorporating ResNet-inspired blocks is not entirely novel, this work stands out due to its emphasis on lightweight design and its ability to efficiently process large input images. Residual connections inspired by ResNet are introduced to enhance gradient flow and mitigate vanishing gradient issues, allowing the model to extract deeper and more nuanced features without significantly increasing computational overhead. The reduction in trainable parameters is another critical aspect of the lightweight design. The original VGG16 architecture contains over 138 million parameters, making it resource-intensive and unsuitable for real-time applications in resource-constrained environments. By selectively integrating residual blocks and optimizing the number of filters in later layers, the modified architecture achieves a significant reduction. The computational complexity of the model, measured in floating-point operations (FLOPs), is reduced to 3.5 GFLOPs, ensuring efficiency for real-time deployment in settings such as sports clinics and mobile diagnostic systems. Another distinctive feature of this work is the ability to handle large input images with dimensions of 512 × 512 pixels. The use of large input images is critical for detecting subtle morphological changes in coronary lesions, which are essential for distinguishing between mild, moderate, and severe severities. Unlike traditional approaches that downsample images to reduce computational load—potentially losing critical diagnostic information—the proposed model retains the original resolution. To achieve this, an additional convolutional layer with a 1 × 1 kernel is introduced to process the single-channel grayscale images into a three-channel format, aligning with the VGG16 architecture’s input requirements. This ensures that the model preserves spatial dimensions and maximizes feature extraction from high-resolution images. The combination of these modifications results in a model that balances computational efficiency and diagnostic accuracy. The lightweight design, coupled with the ability to process large images, makes the proposed model particularly suitable for real-time CAD detection in athletes, addressing the specific needs of this high-risk population.

The proposed model extensively uses the CADICA dataset, focusing on key performance metrics to assess its diagnostic capabilities. The primary metrics include accuracy, recall, precision, and AUC-ROC, which are crucial for ensuring the model reliability in detecting subtle variations in coronary lesions. From a computational perspective, the lightweight design significantly reduces resource requirements compared to SOTA architectures. The proposed model contains only 1.2 million trainable parameters and requires 3.5 GFLOPs, making it suitable for real-time deployment in resource-constrained settings, such as mobile diagnostic systems or sports clinics. Despite processing high-resolution input images, the model maintains computational efficiency without compromising diagnostic performance. These performance metrics form the foundation for the analysis in subsequent sections, highlighting the balance between diagnostic accuracy, computational efficiency, and suitability for real-world applications.

**Figure 1:**
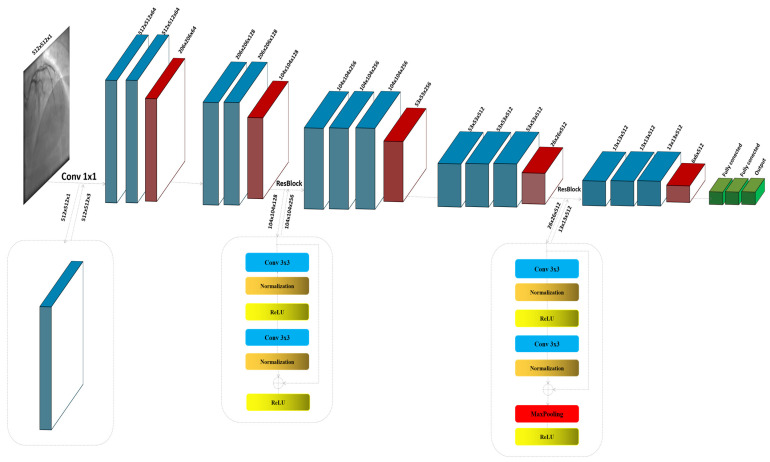
The detailed architecture of the proposed model by modifying VGG16.

The ResNet block, known for its use of residual connections, enables deeper networks by allowing gradients to flow through the architecture more efficiently, thus reducing the problem of vanishing gradients in deep networks. ResNet blocks utilize skip connections, or shortcuts, to jump over some layers. Typical ResNet blocks include two main convolutional layers along with Batch Normalization and ReLU activation functions. The key feature is the addition of the input of the block to its output, usually through element-wise addition, before applying the final ReLU activation. This approach allows the network to learn identity functions as needed, ensuring that deeper layers can still learn effectively even when additional layers do not contribute to better performance. The process commences with the input image $X_{input}\in R^{W\times H\times 1}$, specified as a grayscale image with dimensions of 512 × 512 pixels and a single channel (C = 1). Given the initial design of the architecture for three-channel (RGB) inputs, an additional convolutional layer is strategically integrated at the outset to transition the input from a single-channel to a multi-channel format. This adjustment is crucial as it aligns the input with the expectations of the model, enabling the utilization of robust feature extraction capabilities of the baseline. The convolutional layer, described in Figure 1, employs a kernel size of 1 × 1; this choice is deliberate, ensuring that the spatial dimensions of the image are preserved while the depth is adjusted to three channels, as shown in Figure 1:

(1) $$F_{1\_l}=F_{1\times 1}(X_{input})$$

In this context, the initial convolutional layer, denoted as $F_{1\_l}$, employs a 1 × 1 kernel size specifically designed to modify the number of channels in the input image:

(2) $$F_{blok1}=({MaxPooling}_{2\times 2}{(F}_{conv_{3}\times 3}(F_{conv_{3}\times 3}(F_{1_{f}}))))$$

In the modified architecture, the first block, denoted as $F_{blok1}$, is instrumental in capturing initial feature representations from the feature map $F_{1\_f}\in R^{512\times 512\times 3}$. This block comprises two convolutional layers, each designed to extract pertinent features from the input. Following these convolutional layers, a max pooling operation is employed to achieve dimension reduction:

(3) $$F_{blok2}=({MaxPooling}_{2\times 2}{(F}_{conv_{3}\times 3}(F_{conv_{3}\times 3}(F_{block1}))))$$

$F_{blok2}$ has the same structure and functions like $F_{blok1}$. This sequential arrangement not only enhances the extraction of relevant features but also efficiently reduces the spatial size of the feature maps, thereby decreasing the computational load for layers and improving the overall efficiency of the network. This configuration is fundamental in setting the stage for deeper and more complex feature extraction in later stages of the network, enabling it to discern more nuanced characteristics of the input data:

(4) $$F_{res\_block1}=F_{1}{,F}_{2}$$

$$F_{1}={max(0,(BatchNormalization(F}_{conv\_3\times 3}(F_{block2}))))$$

$$F_{2}=max(0,(BatchNormalization({F_{conv_{3}\times 3}(F}_{1})+F_{block2})))$$

The architecture of the residual block we thoughtfully partitioned into two distinct sections, $F_{1}$ and $F_{2}$, to simplify the understanding of its operations. In the first section, $F_{1}$, the convolution layer acts on the input feature map to extract initial information. This process is immediately followed by batch normalization, which stabilizes and normalizes the activations across the mini-batch, facilitating a smoother training process and improving the convergence efficiency of the proposed model. Subsequent to normalization, the ReLU activation function is applied. This activation function introduces non-linearity to the process, enhancing the ability to capture complex patterns in the data. The second section, $F_{2}$, focuses on the integration of the residual connection. Here, the output from $F_{1}$ is added back to the original input feature map before it passes through $F_{1}$, assuming there is no dimension mismatch. This step is crucial, as it helps to mitigate the problem of vanishing gradients in deep neural networks by allowing gradients to flow directly through the network. Following the residual addition, the result undergoes another ReLU activation. This final application of ReLU ensures that any negative values are zeroed out, maintaining the non-linearity in the activation map which is essential for learning more complex functions. By structuring the residual block into these two parts, $F_{1}$ and $F_{2}$, the model not only becomes easier to analyze and understand but also leverages the strengths of both deep learning and residual learning through direct feature reuse, thereby enhancing overall network performance and learning stability:

(5) $$F_{blok3}=({MaxPooling}_{2\times 2}{(F_{conv_{3}\times 3}(F}_{conv_{3}\times 3}(F_{conv_{3}\times 3}(F_{res_{block}})))))$$

(6) $$F_{blok4}=({MaxPooling}_{2\times 2}{(F_{conv_{3}\times 3}(F}_{conv_{3}\times 3}(F_{conv_{3}\times 3}(F_{block})))))$$

Both blocks in the architecture share a similar structure, with an added convolutional layer in each block for applying an increased number of filters. This enhancement allows for more detailed and nuanced feature extraction from the input data:

(7) $$F_{res\_block2}=F_{1}{,F}_{2}{,F}_{3}$$

$$F_{1}={max(0,(BatchNormalization(F}_{conv\_3\times 3}(F_{block4}))))$$

$$F_{2}=BatchNormalization({F_{conv\_3\times 3}(F}_{1}))+F_{block4}\;$$

$$F_{3}=max(0,MaxPooling(F_{block4}))$$

The second residual block maintains the same configuration of convolutional, normalization, and pooling layers as the first block $F_{res\_block1}$. The notable distinction lies in the pooling function of the layers, which is specifically utilized in the second block to reduce the dimensionality of the feature map $F_{blok4}\in R^{WxHxC}$, enhancing the efficiency of the model by decreasing spatial size while preserving critical features:

(8) $$F_{blok5}=({MaxPooling}_{2\times 2}{(F_{conv_{3}\times 3}(F}_{conv_{3}\times 3}(F_{conv_{3}\times 3}(F_{res_{block2}})))))$$

(9) $$F_{output}=F_{fc1}{,F}_{fc2}{,F}_{fc3}$$

The architecture concludes with three sequential layers designed to flatten the multi-dimensional feature maps into a 1D vector, facilitating dense connections. These dense layers are followed by a Sigmoid activation function, which outputs a binary classification indicating ‘lesion’ or ‘non-lesion’.

## 4. Experiment and Results

The proposed scheme was evaluated using carefully selected parameter settings to optimize performance and ensure robust experimentation. The parameters were chosen based on prior research and fine-tuning during preliminary experiments. Table 1 summarizes the key parameter settings used in the experiments, along with their respective assumptions and rationale.

These parameter settings reflect the trade-off between computational efficiency and diagnostic performance required for CAD detection in athletes. Early stopping criteria were applied to avoid overfitting, and data augmentation ensured robustness in real-world clinical scenarios. The rationale behind each parameter was derived from previous research and tailored to the unique requirements of the CADICA dataset.

### 4.1. The CADICA

**Figure 2:**
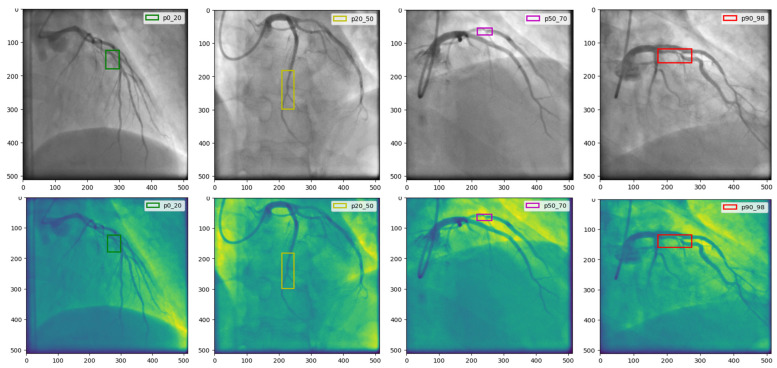
The CADICA dataset is visually segmented into categories based on lesion severity in coronary arteries. The first column displays ‘Mild’ cases with lesions ranging from 0% to 20%, followed by the second and third columns showcasing ‘Moderate’ cases with lesions between 20% and 70%. The last column illustrates ‘Severe’ cases, where lesions range from 90% to 99%.

Our research utilizes the CADICA dataset, specifically curated to assess CAD in athletes who engage in high-intensity endurance sports. This dataset comprises 3000 grayscale images with a resolution of 512 × 512 pixels, representing a balanced mix of lesion severities in coronary arteries. The images are categorized into three severity classes such as, ‘Mild’ in range [0–20% occlusion], ‘Moderate’ in range [20–70% occlusion], and ‘Severe’ in range [70–100% occlusion], with 1000 images per category Figure 2.

Given the dataset’s limited diversity, with images derived from only 42 patients, a patient-level data split was employed instead of an image-level split. This approach ensured that no images from the same patient were included in more than one subset, thus eliminating the risk of information leakage and providing a realistic evaluation of the model’s generalization capabilities. The dataset was divided into three subsets: 70% of the images were allocated to the training set, 15% to the validation set, and the remaining 15% to the test set. Images were distributed such that each subset contained data from entirely distinct patients. This partitioning strategy simulated real-world scenarios where a model encounters previously unseen patients during deployment. To enhance the robustness of the evaluation, 5-fold cross-validation was performed, where patient subsets were rotated across training, validation, and test sets. This ensured that the performance metrics reflected the model ability to generalize across different patient characteristics. To mitigate the effects of the relatively small dataset size, extensive data augmentation techniques were applied to the training set. These included spatial transformations such as rotations, scaling, and flipping, as well as elastic deformations to simulate real-world imaging variations. These augmentations preserved the biological plausibility of the images while improving the model’s ability to handle variations in imaging conditions, such as differences in angle, lighting, and anatomical diversity.

**Figure 3:**
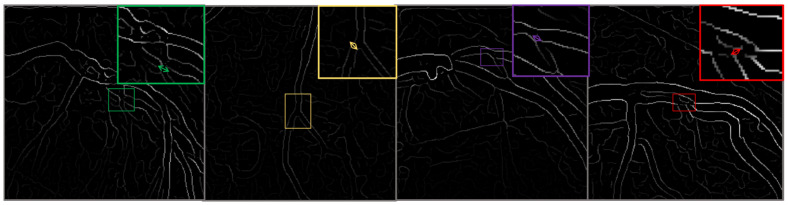
This figure illustrates the outcomes of the image preprocessing steps applied to the feature map, utilizing edge enhancement filters to emphasize the most relevant edges effectively.

This classification facilitates a targeted analysis of the ability of the model to detect and classify lesions accurately across a spectrum of cases. To enhance the robustness of the dataset and to simulate real-world scenarios that may affect the imaging process, such as varying angles and lighting conditions, we augment the images using techniques such as rotation, scaling, and elastic deformations. Each image undergoes preprocessing to standardize the input and improve model efficacy, Figure 3.

We use the CADICA to provide a comprehensive understanding of how variations in coronary artery conditions manifest in high-resolution images. This understanding is pivotal for training the modified VGG16 model to detect subtle nuances that distinguish between different levels of CAD severity. The detailed annotations accompanying each image, verified by a team of expert radiologists, ensure the reliability of the ground truth used in model training and evaluation (Table 2).

#### Strategies for Managing Dataset Limitations and Ensuring Robust Model Evaluation

The CADICA dataset, while offering a substantial collection of angiographic images, poses significant challenges due to its limited patient diversity, with only 42 patients contributing 1000 images. This limitation amplifies the risk of overfitting and information leakage, necessitating stringent measures to ensure robust model evaluation and reliable generalization. To address these challenges, the dataset was partitioned at the patient level rather than the image level, as seen in Table 3. This approach ensured that no images from the same patient appeared across training, validation, and test subsets, thereby eliminating any risk of information leakage that could artificially inflate performance metrics. This splitting strategy was critical to simulate real-world scenarios where the model encounters entirely unseen patients. To mitigate overfitting arising from the relatively small dataset size, extensive data augmentation techniques were employed.

These included spatial transformations such as rotations, flips, and scaling, alongside adjustments to brightness and contrast, as well as elastic deformations that mimic real-world imaging variability. These augmentations enhanced the diversity of the training data while preserving its biological plausibility, thus encouraging the model to learn robust and generalizable features. Cross-validation was performed at the patient level to further evaluate the model performance. This involved partitioning the dataset into multiple folds, ensuring that each fold contained images from distinct subsets of patients. This methodology provided a comprehensive assessment of the model ability to generalize across varying patient characteristics, while also minimizing the risk of bias associated with a single data split. Regularization techniques, including dropout layers and weight decay, were incorporated into the model architecture to reduce the risk of overfitting. Additionally, the use of early stopping based on validation performance ensured that the training process ceased once the model performance began to degrade, preventing over-optimization of the training data. Finally, a completely independent test set was held out from the initial data split. This test set, containing images from a distinct group of patients, was used exclusively for final performance evaluation. This approach ensured that the reported metrics reflected the model real-world diagnostic capabilities rather than its ability to recall patterns from the training or validation sets. By implementing these measures, the study prioritized the robustness and reliability of the proposed model, mitigating the risks associated with dataset limitations and ensuring the validity of the experimental findings.

### 4.2. Comparison of the Results of the Experiment and Analyses

**Figure 4:**
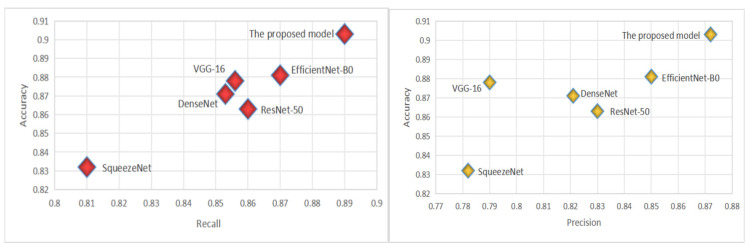
The virtualization of Recall and Precision.

The CADICA dataset provides angiogram images, which are instrumental in diagnosing CAD. These images, combined with the performance data of various deep learning models, offer an insightful exploration into the capabilities of these technologies in medical diagnostics, particularly in the detection of CAD. The models we evaluate by including well-known architectures such as ResNet-50, VGG-16, DenseNet121, SqueezeNet, and EfficientNet-B0, alongside the proposed model. Each performance of the proposed model is meticulously analyzed through three critical metrics such as Accuracy, Recall, and Precision, each vital for ensuring the reliability and effectiveness of medical diagnoses. Accuracy, a primary measure, quantifies overall effectiveness of each model in making correct predictions across all test cases. It reflects the capability of each model to correctly identify both the presence and absence of disease, providing a holistic view of its diagnostic accuracy. The proposed model demonstrates an exceptional accuracy rate of 90.3%, indicating that it correctly predicts the outcomes nearly 91% of the time, which is significantly higher than the other models evaluated, as shown in Table 4. The proposed model achieves a recall rate of 89%, showcasing its robust capability to detect true positives effectively. A high precision rate is essential in reducing the number of false positives, which can lead to unnecessary anxiety and invasive procedures for patients. At a precision rate of 87.2%, the proposed model confirms its reliability in its positive classifications, making it a dependable tool in the clinical decision-making process; see Figure 4.

By employing such advanced deep learning models, healthcare practitioners can leverage the significant improvements in diagnostic accuracy, ensuring that patients receive the most informed and effective care. This comprehensive approach, which combines rigorous statistical analysis with practical visual validation, marks a significant advancement in the use of artificial intelligence in healthcare, paving the way for more precise and dependable medical diagnostics.

### 4.3. Comparison Results with State-of-the-Art Model

To evaluate the effectiveness of our proposed model in detecting and CAD, we conducted extensive experiments comparing its performance against several state-of-the-art (SOTA) deep learning architectures. The models selected for comparison include Refs. [27,28,29,32,33]. These architectures were chosen based on their prominence in medical imaging research and their diverse design philosophies, ranging from residual connections to efficient parameter utilization. The performance evaluation focused on multiple critical aspects: overall accuracy, sensitivity in detecting true positive cases, precision in minimizing false positives, and the balance between these measures as reflected by the F1-score. Furthermore, the ability of the models to distinguish between diseased and non-diseased cases across different thresholds was assessed through the Area Under the ROC Curve (AUC-ROC). Computational complexity, encompassing the number of trainable parameters and Floating Point Operations Per Second (FLOPs), was also examined to ensure practicality in real-world applications.

Table 4 summarizes the performance of the models on the CADICA [28] dataset. The proposed model demonstrates superior results across all major metrics, highlighting its effectiveness in CAD detection.

**Table 4:** The Comparison of Models.

Model	Accuracy	Recall	Precision
ResNet-50	0.863	0.86	0.83
VGG-16	0.878	0.856	0.79
DenseNet121	0.871	0.853	0.821
SqueezeNet	0.832	0.81	0.782
EfficientNet-B0	0.881	0.87	0.85
The proposed model	0.903	0.89	0.872

**Figure 5:**
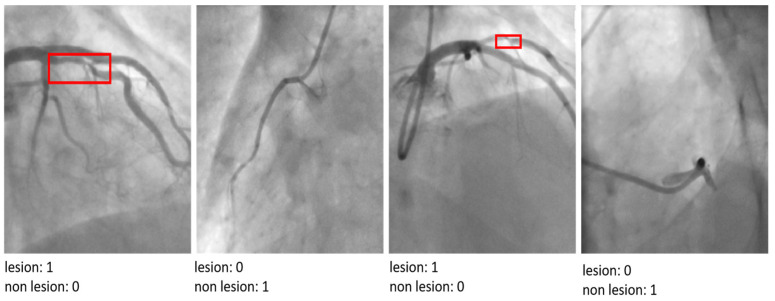
The results of the proposed model by indicating ‘lesion’ or ‘non-lesion’.

Table 5 compares the proposed model performance against several state-of-the-art models referenced in [27] through [33]. The evaluation is based on key performance metrics, including accuracy, recall, precision, F1-Score, and AUC-ROC, as well as computational parameters like the number of trainable parameters (in millions) and the computational complexity measured in FLOPs. The proposed model outperforms all the referenced models across most metrics. It achieves the highest accuracy of 0.903, recall of 0.890, and precision of 0.90, reflecting its ability to correctly identify true positive cases while minimizing false positives. The F1-Score, which balances precision and recall, is also the highest at 0.90, showcasing the model’s overall robustness. Additionally, the AUC-ROC of 0.912 indicates superior discriminative power compared to other models. From a computational perspective, the proposed model is notably efficient. It has the lowest number of parameters (1.2 million) and relatively low computational complexity (3.5 GFLOPs), making it significantly lighter than models like Ref. [28] with 138.4 million parameters and 15.3 GFLOPs. This efficiency ensures that the model is suitable for real-time applications and resource-constrained environments, without compromising performance. The table highlights the proposed model’s ability to achieve state-of-the-art results in accuracy, recall, and AUC-ROC while maintaining computational efficiency, underscoring its suitability for practical deployment in CAD diagnostics (Figure 5).

The visual analysis of classification outputs further corroborates the superior performance of the proposed model. It demonstrated exceptional precision in distinguishing between mild, moderate, and severe lesions, even in cases where other architectures struggled to localize critical features accurately. This ability to analyze and interpret nuanced patterns in angiographic images underscores the utility of the proposed model in addressing the unique challenges of CAD diagnosis in athletes. The proposed model’s performance highlights its capacity to advance CAD detection and classification by integrating advanced architectural innovations. By achieving a robust balance between diagnostic accuracy and computational efficiency, the model represents a significant step forward in the development of AI-driven tools for cardiovascular healthcare.

## 5. Discussion

This study presents a novel approach to detecting and classifying CAD among athletes engaged in high-intensity endurance sports, a population that faces unique cardiovascular risks. By modifying the VGG16 architecture to incorporate ResNet-inspired residual connections, the proposed model addresses key challenges in CAD diagnosis, including the need for accurate detection of subtle lesion morphologies and computational efficiency suitable for real-time applications. The results demonstrate that the proposed model outperforms state-of-the-art (SOTA) architectures such as ResNet-50, DenseNet121, and EfficientNet-B0 in key performance metrics, including accuracy, recall, and AUC-ROC. This highlights the model’s robustness in distinguishing between mild, moderate, and severe lesion severities, even in a high-risk population like athletes. The integration of residual connections enhances the gradient flow and mitigates the vanishing gradient problem, enabling deeper and more effective feature extraction. This architectural innovation is particularly beneficial for identifying subtle variations in angiographic images, which are often challenging to detect manually or using standard deep learning models. One of the critical contributions of this study is the computational efficiency of the proposed model. With only 1.2 million parameters and 3.5 GFLOPs, the model is significantly lighter than conventional architectures, making it suitable for deployment in resource-constrained environments such as sports clinics or mobile diagnostic systems. This efficiency does not compromise diagnostic performance, suggesting that lightweight architectures can achieve SOTA results when appropriately designed. The findings also underscore the importance of tailoring diagnostic tools to the specific needs of the target population. Athletes, due to their high cardiovascular fitness, often present atypical CAD symptoms or remain asymptomatic until severe complications arise. The ability of the proposed model to detect early-stage CAD in this demographic highlights its potential as a valuable tool for preventative healthcare in sports medicine. Despite these promising results, several limitations must be acknowledged. First, the CADICA dataset, while comprehensive, focuses primarily on angiographic images, which may not fully represent the diversity of imaging modalities used in clinical practice. Future research should validate the model’s performance across broader datasets, including multimodal imaging techniques such as CT and MRI. Second, although the model demonstrates exceptional computational efficiency, further testing on edge devices is necessary to confirm its real-time applicability in diverse operational settings. Finally, explainability remains a critical factor for clinical adoption. While the proposed model achieves high accuracy, integrating explainable AI (XAI) mechanisms would enhance trust and usability among healthcare professionals. The implications of this study extend beyond CAD diagnosis in athletes. The methodology and findings provide a framework for developing lightweight, high-performance deep learning models that can be adapted to other medical imaging applications. By addressing key challenges such as accuracy, efficiency, and population-specific requirements, this research contributes to advancing AI-driven personalized healthcare.

The proposed model offers a significant advancement in the field of cardiovascular diagnostics, particularly for the detection of CAD in athletes. By combining diagnostic accuracy with computational efficiency, the model represents a step forward in creating accessible and reliable diagnostic tools tailored to the needs of high-risk populations. Future work should focus on expanding dataset diversity, optimizing deployment, and enhancing model interpretability to ensure broader clinical impact.

## 6. Conclusions

This study presents a novel approach to the detection and classification of CAD through a modified VGG16 architecture tailored for analyzing angiographic images. By integrating residual connections inspired by ResNet, the proposed model effectively addresses the limitations of traditional convolutional neural networks, such as vanishing gradients, while enhancing feature extraction and classification performance. The incorporation of these architectural improvements ensures both high diagnostic accuracy and computational efficiency, making the model well-suited for real-world applications. The experimental evaluation, conducted on the CADICA dataset, highlights the superiority of the proposed model over state-of-the-art architectures such as ResNet-50, DenseNet121, and EfficientNet-B0. The model achieved the highest accuracy (90.3%), recall (89%), and AUC-ROC (0.912) while maintaining a significantly lower parameter count (1.2 M) and computational complexity (3.5 GFLOPs). These results underscore the model’s ability to balance diagnostic performance with resource efficiency, addressing the critical needs of medical diagnostics in resource-constrained environments. Beyond quantitative metrics, the model demonstrated exceptional qualitative capabilities in distinguishing varying severities of CAD, including subtle morphological differences in angiographic images. This precision positions the proposed model as a reliable tool for aiding healthcare professionals in early detection and accurate classification of CAD, particularly in high-risk populations such as athletes engaged in high-intensity sports. The findings of this research pave the way for future advancements in AI-driven healthcare diagnostics. Potential directions for future work include expanding the model validation across larger and more diverse datasets, integrating explainability mechanisms to enhance clinical adoption, and optimizing the model for deployment on edge devices. By bridging the gap between cutting-edge deep learning methods and practical clinical applications, this study contributes to the broader goal of improving patient outcomes through early and accurate disease detection.

## Figures and Tables

**Table 1:** Parameter Settings and Assumptions for Experimentation.

Parameter	Value/Setting	Assumptions/Rationale
Input Image Size	512 × 512 pixels	Large images ensure detailed feature extraction of coronary lesions while preserving diagnostic information.
Learning Rate	0.001	A low learning rate stabilizes training and prevents overshooting the optimal solution.
Batch Size	16	Selected to balance memory efficiency and gradient estimation stability.
Optimizer	Adam	Chosen for its ability to adapt learning rates dynamically for faster convergence.
Loss Function	Binary Cross-Entropy	Suitable for binary classification tasks like lesion detection (lesion vs. non-lesion).
Epochs	50	Early stopping was applied to terminate training if validation performance plateaued.
Weight Initialization	He Initialization	Optimized for deep networks with ReLU activation functions to address vanishing gradients.
Data Augmentation	Rotation, Scaling, Flipping, Elastic Deformations	Enhances model robustness to real-world variations in imaging conditions.
Cross-Validation	5-fold Patient-Level Split	Ensures robust evaluation by preventing information leakage and simulating real-world scenarios.
Number of Residual Blocks	2	Balances model depth and computational efficiency to maintain lightweight architecture.

**Table 2:** The Used Metrics.

Metric	Formulation Description
Accuracy	$\mathrm{Accuracy}=\frac{TP+TN}{TP+TN+FP+FN}$
Sensitivity (Recall)	$\mathrm{Sensitivity}=\frac{TP}{TP+FN}$
Specificity	$\mathrm{Specificity}=\frac{TN}{TN+FP}$
Precision	$\mathrm{Precision}=\frac{TP}{TP+FP}$
F1-Score	$\frac{Precision\;x\;Recall}{Precision+Recall}$
Area Under the ROC Curve (AUC-ROC)	Area under the curve of the Receiver Operating Characteristic; higher values indicate better performance.
Number of parameters	The number of parameters in a neural network indicates the total count of trainable weights and biases in the model.
Floating Point Operations Per Second FLOPs	FLOPs are calculated to understand the computational complexity of processing a single input through the network.

**Table 3:** Summary of Strategies to Address Dataset Challenges and Ensure Robustness.

Challenge	Solution	Description
Risk of Information Leakage	Patient-level data split	The dataset was split by patients, not images, ensuring no overlap of patient data across training, validation, and test sets. This approach eliminates the possibility of the model learning patient-specific features that could bias results.
Overfitting	Extensive data augmentation	Applied spatial (rotation, flipping, scaling), color (brightness, contrast), and elastic deformation augmentations to increase diversity in the training set while maintaining biological plausibility.
Dataset Size and Diversity	Cross-validation at patient level	Performed k-fold cross-validation with patient-level splits, ensuring that each fold used distinct patient data. This robust evaluation method provided insights into model generalization across different patient subsets.
Model Complexity	Regularization techniques (dropout, weight decay)	Integrated dropout layers and weight decay to constrain the model and reduce the risk of overfitting on the small dataset.
Training Efficiency	Early stopping	Monitored validation loss and implemented early stopping to terminate training once the model’s performance began to degrade, preventing over-optimization.
Evaluation Reliability	Independent test set	Reserved a separate subset of patient data exclusively for final testing. This ensured that the test set remained unseen during training and validation, providing an unbiased evaluation of the model’s diagnostic performance.
Feature Robustness	Explainable AI tools as Grad-CAM	Used interpretability tools to confirm that the model relied on relevant image features and not spurious patterns or artifacts, ensuring the model learned clinically meaningful representations.

**Table 5:** Comparative Analysis of CAD Detection Models on Key Metrics.

Model	Accuracy	Recall	Precision	F1-Score	AUC-ROC	Parameters (M)	FLOPs (G)
Ref. [27]	0.87	0.87	0.82	0.85	0.88	23.6	4.1
Ref. [28]	0.87	0.86	0.80	0.83	0.89	138.4	15.3
Ref. [29]	0.87	0.86	0.83	0.84	0.89	7.98	2.8
Ref. [32]	0.84	0.82	0.81	0.80	0.87	1.3	0.8
Ref. [33]	0.88	0.88	0.86	0.86	0.91	5.3	0.4
**Proposed Model**	**0.903**	**0.890**	**0.90**	**0.90**	**0.912**	**1.2**	**3.5**

## Data Availability

The original contributions presented in this study are included in the article. Further inquiries can be directed to the corresponding authors.
